# Optimized Stoichiometry for CuCrO_2_ Thin Films as Hole Transparent Layer in PBDD4T-2F:PC_70_BM Organic Solar Cells

**DOI:** 10.3390/nano11082109

**Published:** 2021-08-19

**Authors:** Lorenzo Bottiglieri, Ali Nourdine, Joao Resende, Jean-Luc Deschanvres, Carmen Jiménez

**Affiliations:** 1 French National Centre for Scientific Research, Laboratoire des Matériaux et du Génie Physique, Institute of Engineering, Université Grenoble Alpes, 38400 Grenoble, France; jean-luc.deschanvres@grenoble-inp.fr (J.-L.D.); carmen.jimenez@grenoble-inp.fr (C.J.); 2 French National Centre for Scientific Research, The Laboratory of Electrochemistry and Physical-Chemistry of Materials and Interfaces, Institute of Engineering, Université Grenoble Alpes, University of Savoy Mont Blanc-Chambery, 38000 Grenoble, France; ali.nourdine@univ-smb.fr; 3 AlmaScience Colab, Madan Parque, 2829-516 Caparica, Portugal; joao.resende@almascience.pt

**Keywords:** organic photovoltaics, organic solar cells, OPV, transparent conductive oxides, PEDOT:PSS substitution, out of stoichiometry CuCrO_2_ thin films, hybrid solar cells, metal oxides based HTL, material stability issues and remedies

## Abstract

The performance and stability in atmospheric conditions of organic photovoltaic devices can be improved by the integration of stable and efficient photoactive materials as substituent of the chemically unstable poly (3,4-ethylene dioxythiophene):polystyrene sulfonate (PEDOT:PSS), generally used as organic hole transport layer. Promising candidates are p-type transparent conductive oxides, which combine good optoelectronic and a higher mechanical and chemical stability than the organic counterpart. In this work, we synthesize Cu-rich CuCrO_2_ thin films by aerosol-assisted chemical vapour deposition as an efficient alternative to PEDOT:PSS. The effect of stoichiometry on the structural, electrical, and optical properties was analysed to find a good compromise between transparency, resistivity, and energy bands alignment, to maximize the photovoltaic performances., Average transmittance and bandgap are reduced when increasing the Cu content in these out of stoichiometry CuCrO_2_ films. The lowest electrical resistivity is found for samples synthesized from a solution composition in the 60–70% range. The optimal starting solution composition was found at 65% of Cu cationic ratio corresponding to a singular point in Hackee’s figure of merit of 1 × 10^−7^ Ω^−1^. PBDD4T-2F:PC_70_BM organic solar cells were fabricated by integrating CuCrO_2_ films grown from a solution composition ranging between 40% to 100% of Cu as hole transport layers. The solar cells integrating a film grown with a Cu solution composition of 65% achieved a power conversion efficiency as high as 3.1%, representing the best trade-off of the optoelectronic properties among the studied candidates. Additionally, despite the efficiencies achieved from CuCrO_2_-based organic solar cells are still inferior to the PEDOT:PSS counterpart, we demonstrated a significant enhancement of the lifetime in atmospheric conditions of optimal oxides-based organic photovoltaic devices.

## 1. Introduction

Organic solar cells (OSC) have attracted wide interest in the last decades, because they combine photo-activity, limited cost, and the potential large-scale manufacturing [1], with an adequate Power Conversion Efficiency (PCE) as high as 17.3% [2] (certified). In organic photovoltaic (OPV) devices, the absorber is inserted between the Hole Transport Layer (HTL) and the Electron Transport Layer (ETL). Classically, these solar cells contain poly (3,4-ethylene dioxythiophene):polystyrene sulfonate (PEDOT:PSS) as HTL [3,4]. This polymeric material has been selected due to its solubility in water [5], which allows its synthesis through solution-based processes, and to its high transparency, around 90%. However, this polymer presents several disadvantages such as heterogeneous electrical properties [6], inefficient electron blocking function as proved by results from polymer light-emitting devices [7], with a Lowest Unoccupied Molecular Orbital (LUMO) at 3.5 eV [8,9] and a Highest Occupied Molecular Orbital (HOMO) around 5.2 eV [10,11,12]. This polymer is also characterized by a strong acidity, which promotes the corrosion of the Indium Tin Oxide (ITO) layer used as transparent electrode [13,14,15]. Furthermore, PEDOT:PSS shows limited application because the atmospheric humidity and oxygen intake alter its performances [16], which will cause a severe reduction of the photogenerated current [17], and, finally, a diminished lifetime of the devices [18].

In this paper, we aim to tackle the replacement of unstable PEDOT:PSS. An alternative is fundamental for the conception of performant and stable in atmospheric condition OSCs. Candidate materials for this purpose have to satisfy specific requirements [19,20,21] such as (i) high p-type conductivity to enhance the charge collection at the anode (ii) good electron blocking function with energetic levels compatible with those of the adjacent layers, thus efficiently working as electron blocking layer (EBL) [21], (iii) adequate transparency, allowing the greatest number of photons to impinge over the active layer and, thus, participate to the photogeneration [22] and (iv) chemical stability in atmospheric conditions and neutrality to the adjacent layers [21]. To fabricate OSCs reliably in atmospheric conditions, a promising solution is represented by a hybrid combination of organic and inorganic materials, integrating p-type transparent conductive oxides (TCOs) as HTL, elaborated using a reproducible synthesis route by solution-based processes at relatively low temperatures. Among them, Cu^+1^-based delafossites (Cu^+1^M^+3^O_2_) arose as novel promising candidates for this application because of their relatively high p-type conductivity (0.1–1 S cm^−1^), an appropriate bands’ alignment and good transparency in the visible. Furthermore, it is well known that these materials are stable in atmospheric conditions [23,24]. For instance, CuGaO_2_ nanoplates have been reported as possible candidates for photovoltaic applications [25]. However, the large size of the CuGaO_2_ nanoparticles limits the maximum achievable performances [25]. Based on this consideration, the selection of a different delafossite material is required. In particular, CuCrO_2_, with its high transparency, hole conductivity, and UV absorption, emerges as promising HTL, which is supposedly able to increase the performances and the stability in atmospheric conditions of the photovoltaic devices [26,27]. Other p-type TCOs were successfully integrated as HTL in OSC such as MoO_x_ [28], and NiO_x_ [21]. On one hand, it has been demonstrated that, for a given active layer, the use of PEDOT:PSS, CuCrO_2_ or MoO_x_, would not lead to significant differences in photovoltaic performances [22]. On the other hand, nickel oxide presents a high light absorption in the visible range, small holes’ diffusion coefficient, and carrier mobility that limits the device performance [29,30,31]. Furthermore, it has been reported that the integration of CuCrO_2_ leads to higher efficiencies than the use of NiO_x_ [27]_._ Based on these considerations, CuCrO_2_ was selected as promising HTL in OPV devices. Stoichiometric CuCrO_2_ nanoparticles were successfully implemented as HTL in OSC [32], dye-sensitized solar cells (DSSC) [30], and perovskite solar cells [24]. The integration of this nanomaterial in perovskite photovoltaic devices was reported to improve the lifetime of the cells under illumination and the thermal stability of the device [24]. Despite that it is well-known that the cationic ratio, Cu/(Cu + Cr), plays a major role in the electrical and optical properties of this compound, with the best performances found for non-stoichiometric films [33,34,35,36,37,38,39], there is a lack of studies about the integration of these thin films in solar cells. Wang et al. [40] reported the integration of CuCrO_2_ with an oxygen excess, CuCrO_2+x_, in OPV devices achieving values of PCE as high as 4.63%. Nevertheless, the effect of the cationic ratio over the photovoltaic performances of these devices has not been studied until now.

Based on this analysis, we synthesized CuCrO_2_ thin films by aerosol-assisted chemical vapour deposition (AA-CVD), while tuning the cationic ratio in the initial solution, Cu/(Cu + Cr), between 40 and 100% to modify their structural, electrical, and optical properties. We further demonstrated the optoelectronic properties of these p-type TCOs through the integration as HTL in PBDD4T-2F: PC_70_BM based devices. To our knowledge, this is the first time where the applicability of CuCrO_2_ with various cationic ratios as HTL in PBDD4T-2F:PC_70_BM OSC was proved. Additionally, the stability in atmospheric conditions of these devices was analysed. In this article, we report the successful integration of out of stoichiometry CuCrO_2_ thin films synthesized at low temperature and ambient pressure, in OPV devices. A simple chemical approach is used to tune the properties of the HTL and, linked to the performances of the solar cells. The good efficiencies obtained by the oxide-based solar cells are still inferior to the PEDOT:PSS control device, although the found enhancement of the lifetime of the device in atmospheric conditions is considered a positive result.

## 2. Material and Methods

### 2.1. Deposition of CuCrO_2_ Thin Films

CuCrO_2_ thin films with different stoichiometry were synthesized by aerosol-assisted metal-organic chemical vapour deposition (AA-MOCVD) in a cold wall vertical flux reactor [41], at atmospheric pressure. Further information about the deposition system and the characterization techniques used in this work can be found in a previous published work [33]. The precursor starting liquid solution was composed of copper (II) acetylacetonate (Cu(acac)_2_-CuC_10_H_14_O_4_) and chromium (III) acetylacetonate (Cr(acac)_3_-CrC_15_H_21_O_6_) dissolved in ethanol. The total molar concentration, $\left[Cu{\left(acac\right)}_{2}\right]\;+\;\left[Cr{\left(acac\right)}_{3}\right]$ was of 10 mM. The solubility of the precursors was improved by the addition of ethylenediamine (C_2_H_8_N_2_, Sigma-Aldrich, St. Louis, MO, USA) with a concentration of 20 mM. The cationic molar ratio in the solution, X i.e., $\frac{\left[Cu{\left(acac\right)}_{2}\right]}{\left(\left[Cu{\left(acac\right)}_{2}\right]+\left[Cr{\left(acac\right)}_{3}\right]\right)}$, was varied from 40% to 100%. Alkaline earth boro-aluminosilicate glass (Corning 1737, 2.5 cm × 2.5 cm, ≈1 mm thick) or ITO patterned glass (Ossila S101) were used as substrate. The corning substrates were first cleaned through a brushing step using paper and various solvents: acetone, isopropanol, and deionized water; then they were cleaned by ultrasonication in isopropanol for 20 min, abundantly rinsed with deionized water and dried with clean compressed air. During the deposition, the substrate temperature was set to 350 °C and the deposition duration was 60 min. Dry compressed air was used as process gas with a total flow rate of 4800 sccm. The solution consumption rate was fixed at 2 mL/min. The oxygen partial pressure during the film growth was 0.21 × 10^5^ Pa.

### 2.2. Characterization of Thin Films

The composition of the films was surveyed through energy-dispersive X-ray spectroscopy (EDX) using an FEI Quanta 250 field-emission scanning electron microscope (FESEM) (FEI, Hillsboro, OR, USA) equipped with an Oxford Inca Energy detector. The energy of the beam was set to 15 KeV. The thickness of the films was measured by cross-section observations on an FEG-ZEISS-Gemini 300 scanning electron microscope (SEM) (FEI, Hillsboro, OR, USA). Raman spectra were acquired at room temperature using a Jobin Yvon/Horiba LabRam spectrometer (Horiba, Kyoto, Japan) equipped with a liquid nitrogen-cooled charge-coupled detector (Horiba, Kyoto, Japan). An Argon laser (Horiba, Kyoto, Japan), wavelength of 488 nm, with a spot size around 1 μm^2^ was employed as source and focused by using a 100× magnification lens. The laser power at the sample surface was around 80 µW. X-ray diffraction (XRD) patterns were obtained using a Bruker D8 Advance diffractometer in θ–2θ configuration (Bragg–Brentano) with a Cu K_α1_ radiation source, λ = 0.15406 nm, (Billerica, MA, USA). The surface roughness of the films was measured by atomic force microscopy (AFM) on a Veeco D3100 AFM (Veeco, Plainview, NY, USA) over 1 µm^2^ surface, and the acquired data treated by Gwyddion software (version 2.58, Department of Nanometrology, Czech Metrology Institute, Brno, Czech Republic). Optical measurements were performed by UV-VIS-IR spectroscopy, on a Lambda 950 spectrophotometer (Perkin Elmer, Waltham, MA, USA) from Perkin Elmer equipped with an integrating sphere, using a wavelength step of 5 nm. Electrical properties were measured by linear 4-probe setup with a distance of 1 mm between the tips. The electronic transport properties were measured at room temperature by Hall-effect in a homemade setup with a magnetic field of 0.5 T. 

### 2.3. OSC Fabrication and Characterization

All the devices were fabricated on ITO patterned glass (Ossila S101) provided with a 100 nm thick ITO layer presenting a sheet resistance of 20 Ω/sq and a root mean square (RMS) roughness of 1.8 nm. Substrates were sonicated at room temperature in acetone, ethanol, and isopropanol for 5 min each. Finally, the substrates were rinsed with deionized water. The control devices were fabricated with a 30–40 nm thick PEDOT:PSS layer as HTL. Before PEDOT:PSS deposition, the ITO patterned glasses were cleaned by UV-ozone treatment (Ossila UV ozone cleaner E511) for 35 min. After filtration using glass fibre filters of 0.45 um, 50 µL of commercial PEDOT:PSS suspension in water (Al 4083, Ossila) with a concentration of 1.3 to 1.7 wt.%, resistance range of 500–5000 Ω cm, and work function of 5–5.2 eV was spin-coated using a KLM SCC 200 from Schaefer techniques. The deposition was performed at 83 rps for 30 s, and a drying step after deposition on a hot plate at 120 °C for 20 min allows to obtain a continuous and homogeneous layer of 30–40 nm. When using AACVD CuCrO_2_ films as HTL, the ITO film area was previously divided into several anodes through laser engraving. This low cost and rapid approach permits us to electrically separate the devices with high precision [42]. 

The deposition of the active layer (AL) and the characterization of the solar cells were performed in a controlled atmosphere through the use of a homemade N_2_ filled glove box (Glas-Col-27–27). A hygrometer and an oxygen sensor were used to measure the humidity and the proportion of oxygen in the glove box, respectively. During the experiment, the relative humidity (RH) was lower than 3%, and the oxygen partial pressure varied between 0.2 to 1%. The AL is based on commercial donor PBDD4T-2F low bandgap polymer purchased from Ossila (Sheffield, UK) (138 Kg/mol, PDI = 2.6) and fullerene acceptor PC_70_BM from Solarmer (El Monte, CA, USA) (purity 99%). As well known in OPV devices optimization, the optimal D/PC_70_BM ratio depends mainly on the device architectures, i.e., direct (glass/anode/HTL/AL/ETL/cathode) or inverse (glass/cathode/ETL/AL/HTL/anode) configurations and the processing conditions of the AL that control the size distribution of the donor/acceptor phases between anode and cathode [43,44]. In this work, a direct configuration was used and preliminary tests, not reported here, highlighted a weight ratio PBDD4T-2F:PC_70_BM of 1.5:1 as optimal. The AL solutions were prepared using anhydrous chlorobenzene as solvent and a total concentration, PBDD4T-2F:PC_70_BM, of 10 g/L. The solution was stirred at 80 °C for 2 h. Then, 1,8-diiodooctane (DIO) with a volumetric concentration of 3 vol.%, was mixed as additive co-solvent and the resulting solution was stirred again for 1 h before the deposition of AL. The obtained solution was spin-coated over glass/ITO/PEDOT:PSS and glass/ITO/CuCrO_2_ substrates. Before the AL deposition, the glass/ITO/CuCrO_2_ were exposed to UV-O_3_ treatment for 20 min. The spin-coating process was performed by using two progressive rotation rates first at 25 rps during 30 s with a ramp of 4 s and, secondly, with 80 rps during 30 s with a ramp of 4 s, resulting in a 100 to 120 nm thick active layer. A dynamic methanol washing step at 25 rps for 30 s was performed to remove residual additives in the AL [44]. A drying step of 1 h under vacuum at 10^−6^ mbar was performed to remove the solvents and DIO traces. Preliminary studies revealed a strong impact of this step over the OSC performances, as reported in literature [44,45]. Finally, 1 nm of LiF and 100 nm of Al were sequentially thermally evaporated on top of the active layer to complete the device. The J-V measurements were recorded under A.M. 1.5 standard illumination using a Oriel LCS-100 solar simulator (Newport, North Kingstown, RI, USA) with an irradiation of 100 mW/cm^2^ from the glass side using an Agilent B2902A Source Measure Unit. A variable number of solar cells were studied for each HTL composition, with a device surface of 4.5 mm^2^.

## 3. Results and Discussion

### 3.1. Characterization of Out of Stoichiometry CuCrO_2_ Thin Films

**Figure 1:**
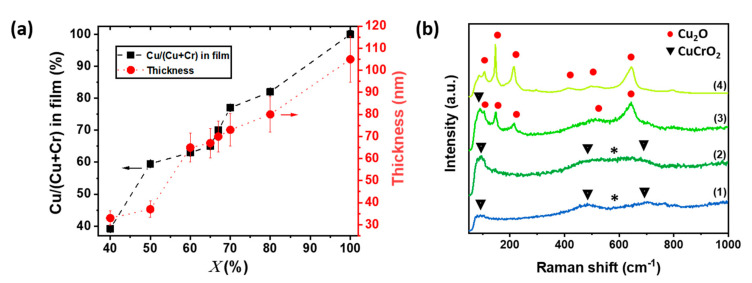
Cationic ratio, thickness, and structural properties of out of stoichiometry CuCrO_2_ thin films. (**a**) Variation as a function of cationic ration in solution, X, of the cationic ratio Cu/(Cu + Cr) in the film (left axis, black squares connected by dashed line) measured by EDX and the thickness of the films measured by SEM cross-section observation (right axis, red circles connected by dotted line). (**b**) Raman spectra for samples deposited using a X value of (1) 40%, (2) 65%, (3) 67%, and (4) 100%.

The detailed analysis of the results obtained on CuCrO_2_ thin films grown on glass substrate intends to understand the relationship between the chemical composition of CuCrO_2_ films, its optoelectronic and end-use properties. We assume that film properties are similar for CuCrO_2_ films deposited on glass and on ITO-patterned glass, so the samples are identified by the Cu content of the precursor solution, X. The cationic ratio in the films, Cu/(Cu + Cr), as determined by EDX and the corresponding thickness obtained from SEM cross-section observations are reported in Figure 1a. The films are Cu-rich compared to the initial solution composition, X, as we previously reported in our work [33]. The thickness of the out of stoichiometry CuCrO_2_ thin films present a large variation dependent on the Cu content, ranging from 35 nm in the X = 40% case up to 105 nm for pure Cu_2_O. The increasing film thickness with higher Cu content can be attributed to the growth mechanism of CuCrO_2_ delafossite phase_,_ in agreement with previous works on this material [33,34,35]. Raman spectroscopy was employed to investigate the structural properties of the out of stoichiometry CuCrO_2_ thin films. The Raman spectra and XRD patterns of all the samples are presented in supporting information (SI), in Figure S1a,b, respectively. The Raman spectra for films deposited from solution concentrations of 40, 65, 67, and 100% of Cu cationic ratio, X, are presented in Figure 1b. Three Raman modes (triangles in Figure 1b) are observed at 96 cm^−1^ (E_u_), 460 cm^−1^ (E_g_), and 709 cm^−1^ (A_1g_), corresponding to the CuCrO_2_ phase [46]. These modes are visible for samples deposited from a solution with X≤65%, spectra (1) and (2) of Figure 1b. Additional modes are detected in the 500–650 cm^−1^, labelled as *, which are attributed to the presence of intrinsic defects, likely Cu vacancies or oxygen interstitial [47]. Their intensity increases with Cu content, suggesting a greater number of defects for X = 65% when compared to X = 40%. For X = 67%, spectra (3) in Figure 1b, the E_u_ mode of the CuCrO_2_ at 96 cm^−1^ is detected, while additional Raman modes, at 108 cm^−1^, 149 cm^−1^, 216 cm^−1^, 495 cm^−1^, and 649 cm^−1^ are attributed to the presence of the Cu_2_O phase [48]. This statement is confirmed by the comparison with the spectrum of pure Cu_2_O deposited in the same deposition conditions (Figure 1b, spectra (4)).

These results are corroborated by the XRD patterns of these samples (Figure S1b). From these crystallographic patterns, we can also be evinced that the relative intensity of the (012) peak increases from X = 60% to X = 65%. This suggests that a higher cationic ratio leads to an improvement of the films’ crystallinity with the formation of larger grains. In agreement with our previous results [33], the films structures can be tuned as single-phase CuCrO_2_ films from X = 40 to 65%, and as a nanocomposite material formed by a mixture of grains of Cu_2_O and CuCrO_2_ for X ≥ 67%. The morphologies of the CuCrO_2_ films were surveyed by AFM. The top view, 3D map, and the values of (RMS) roughness for samples synthesized by an initial solution with X = 40%, 50%, 60%, 65%, 70%, and 100% are reported in Figure S2 in Supplementary Materials. The CVD process allowed the deposition of smooth thin films characterized by RMS roughness values around 1–2 nm. The optical properties were also analysed. The visual aspect of the films was mirror-like. The increase of Cu content in the film changes the colour of the films from light grey for X = 40% to bright yellow for X = 100%. The transmittance spectra of these layers were measured and the results are presented in Figure S3. Increasing the Cu content leads to a reduction of the average transmittance in the visible, 390–700 nm, from around 65% for X = 40% down to 45% for X = 80%. This decrease in transmittance will limit the number of photons able to reach the AL, thus reducing the photogeneration when applied these films as HTL in solar cells. The absorbance of the films was modelled as:

(1) $$\alpha \;=\frac{1}{d}*\ln\left(\frac{1}{T}\right)$$

**Figure 2:**
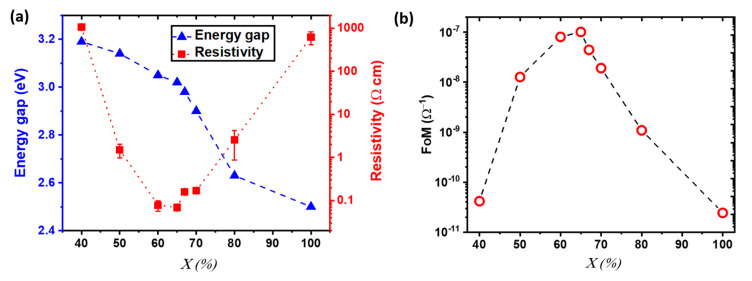
Electrical properties, energy gap, and Figure of Merit for out of stoichiometry CuCrO_2_ thin films. (**a**) Evolution of (left axis) the energy gap (blue triangles) as estimated by Tauc plot, and (right axis) the resistivity values (red square and the relative error is reported as red line) as a function of the copper cationic ratio in the solution, X. (**b**) Hackee’s figure of merit (FoM) as a function of X.

With α the absorbance, d the thickness, and T the transmittance of the thin films. A Tauc plot representation was used to estimate the direct bandgap of the out of stoichiometry CuCrO_2_ thin films, showing a reduction of the optical bandgap for increasing values of X, Figure 2a. The measured values varied in the 3.2 eV and 3.0 eV range, for X = 40% and 65%, respectively, corresponding to the single-phase out of stoichiometry CuCrO_2_ thin films. These values are in agreement with literature [49]. A further decrease of the energy gap is observed for composite materials (X ≥ 67%) likely related to the formation of an additional optical transition associated with the appearance of the Cu_2_O phase, with values as low as 2.5 eV for pure Cu_2_O. As explained by Kawazoe et al. [50], the direct interaction among 3d^10^ electrons in the proximity of Cu^+1^ cations is responsible for the decrease in energy gap. An increasing cationic content would lead to a greater amount of Cu atoms in the film, which would correspond to a stronger interaction between 3d^10^ electrons, finally lowering the bandgap, as reported for Cu_2_O [51]. We also studied the variation of the resistivity with the cationic ratio in the solution, as shown in Figure 2a. It has to be mentioned that in CuCrO_2_, the indirect bandgap influences the electrical properties, while the direct energy gap will impact the optical properties [52]. Thus, electrical resistivity and energy gap do not follow the same trend. The Cu acts as non-intentional doping that leads to a reduction of resistivity from over 1000 Ω cm for X = 40% down to 0.07 Ω cm for X = 65%. The reduction in electrical resistivity for Cu-rich CuCrO_2_ is attributed to two simultaneous effects. On one hand, a higher Cu content would lead to an increase in the number of defects in agreement with the greater intensity of the defects-related Raman modes found for Cu-rich films. Due to the Cu-rich/Cr-poor stoichiometry of our films, we may speculate that the main intrinsic dopants are Cu in antisite defects, i.e., the extra Cu atoms might occupy the Cr vacancies. This is in agreement with previous reports on Cr-deficient CuCrO_2_ [53,54]. On the other hand, we have demonstrated that the Cu excess leads to the synthesis of more crystallized films, as demonstrated by XRD, with a favourable electronic structure [33]. For nanocomposite Cu_2_O + CuCrO_2_ films with X = 67% the electrical performances, with resistivity values around 0.1 Ω.cm, are comparable to the optimal CuCrO_2_. For greater X, the resistivity increases up to 1000 Ω cm for pure Cu_2_O. The increasing values of resistivity found for films with X > 65% can be related to the appearance of a more resistive Cu_2_O phase over the more conductive CuCrO_2_, resulting in increasing electrical resistivity up to the values of single-phase Cu_2_O. The degradation of the electrical properties can be attributed to the modification in grain shape and degradation of the crystallinity of the films through the formation of the secondary phase, as reported in our previous work [33]. For comparison, the PEDOT:PSS reference on corning glass has a resistivity value around 10^16^ Ω cm. When measuring the mobility, it was found that single phase CuCrO_2_ thin films have values inferior to the detection limit of the used setup, 0.1 cm^2^V^−1^s^−1^, thus resulting in unreliable measurement in agreement with literature [34,49,55]. In contrast, nanocomposite thin films of Cu_2_O + CuCrO_2_ show a measurable Hall effect p-type mobility with values of µ = 0.88 cm^2^V^−1^s^−1^ and charge carrier density N = 9.8 × 10^19^ cm^−3^ for X = 70% and µ = 5 cm^2^V^−1^s^−1^ and a N = 1.5 × 10^16^ cm^−3^ for pure Cu_2_O. These characterizations allowed us to confirm the p-type behaviour for these measured samples. The integration in a planar heterojunction, not reported here, composed by CuCrO_2_ thin films with X = 40%, 60%, 65% combined with n-ZnO, showed a rectifying behaviour, thus confirming the p-type conductivity for all the studied compositions. To properly compare the electrical and optical properties of the out of stoichiometry CuCrO_2_ thin films despite the variation in thicknesses, the Figure of Merit (FoM) was calculated following the Haacke’s formula [56]:

(2) $$\mathrm{FoM}\;=\;\frac{T^{10}}{R_{\mathrm{sheet}}}$$

where T is the average transmittance in the visible region (390–700 nm), and R_sheet_ is the sheet resistance value. The variation of the FoM with X is reported in Figure 2b.

Despite this calculation is a proper approach to evaluate the performances of TCOs, it has to be noticed that this definition of FoM privileges transparent films over conductive ones. Moreover, a weak dependence of the transparency with thickness can hinder the quantitative comparison among films for samples with a large variation in thicknesses [57]. In our case, the limited fluctuation in thickness allowed us to assert the validity of this comparison. Furthermore, it is important to mention that the FoM does not take into account important parameters for optoelectronic devices, such as energy gap and carrier mobility of the thin films. 

The variation of the electrical and optical properties leads to a FoM that first increased then diminished with the highest value achieved for X = 65%, around 10^−7^ S, due to the combination between an electrical resistivity of 0.07 Ω cm and an average transmittance in the visible spectrum of 50%.

### 3.2. Integration of CuCrO_2_ Thin Films out of Stoichiometry as HTL in OSC

**Figure 3:**
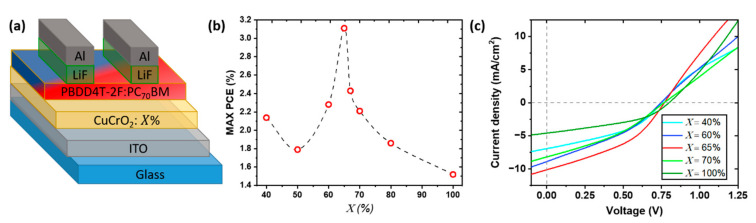
Scheme of the integration of out of stoichiometry CuCrO_2_ thin film as HTL in OSCs based on the active layer of PBDD4T-2F:PC_70_BM 1.5:1. (**a**) Schematic structure of the assembled OSC with out of stoichiometry CuCrO_2_ thin films as HTL, labelled with CuCrO_2_: X. (**b**) PCE variation as a function of X for the best device, and (**c**) corresponding J-V characteristic under A.M. 1.5 illumination. In the latter, not all compositions are presented for higher clarity.

To validate the optoelectronic applicability of the synthesized CuCrO_2_ thin films, we applied these materials as HTL in OSC devices based on standard configuration: glass/anode(ITO)/HTL(PEDOT: PSS or CuCrO_2_)/AL(PBDD4T-2F:PC_70_BM)/ETL(LiF)/cathode(Al). The assembled solar cells are labelled as CuCrO_2_: X, with X representing the cationic ratio Cu/(Cu + Cr) in the starting solution. Thus, the structure of the photovoltaic devices can be listed as glass/ITO/CuCrO_2_: X/PBDD4T-2F:PC_70_BM/LiF/Al, schematically represented in Figure 3a. 

The photovoltaic properties of the devices were confirmed through the measurement of the J-V characteristic under AM 1.5G illumination. For each composition, a variable number of cells was tested to confirm the validity of the procedure. All the devices were analysed to perform a statistical analysis used to corroborate the obtained results, as shown in Figure S4.

The maximum values of PCE for each X values are reported in Figure 3b. This trend is corroborated by the average values, reported in Table 1, and by the statistical analysis of the devices (Figure S4a in Supplementary Materials), confirming the reproducibility of the obtained results. Overall, the PCE increase from 2.15% for CuCrO_2_: 40% up to 3.1% for CuCrO_2_: 65% (Figure 3b). We suppose that the increase in efficiency with X is related to a synergetic effect between the transparency, the conductivity of the HTL, and the band alignment. On one hand, a more transparent film will allow a greater number of photons to reach the active layer, therefore, resulting in a greater photogeneration; from this consideration, an HTL with X = 40%, characterized by the highest average transmittance of this study around 63%, will represent the most suitable candidate for this purpose. Indeed, by increasing X, the optical properties of the films deteriorate, probably due to the deposition of a film with a lower energy gap. On the other hand, a higher HTL conductivity will allow a more efficient charge collection at the anode, increasing the photocurrent. The electrical resistivity is reduced for increasing Cu content, with the lowest resistivity found for X = 65%. Based on these considerations, it seems coherent that the highest PCE was found for CuCrO_2_: 65%, representing the best trade-off between electrical, optical properties and a proper band alignment. The increase in thickness affects optical properties and sheet resistance of the thin films, so the comparison among the different CuCrO_2_ thin film performances as HTL can be hindered. Nevertheless, for Cu-rich thin films, i.e., X between 60% and 80%, the range of thickness variation is reduced to only 15 nm, varying from 65 to 80 nm; therefore, the photovoltaic parameters of OSCs using Cu-rich CuCrO_2_ films as HTL can be properly compared, where all the thin films present a max PCE above 2.0%. An optimization of the thickness of the HTL for several Cu content would be the subject of further studies to establish the influence on PCE values, maybe leading to improved OSC performances.

Another aspect that can be at the origin of the difference in PCE is the superficial roughness of the HTL, which can tune the effective interfacial surface between the AL and the EBL impacting the achievable efficiency of the cell. A rougher layer will lead to an increased contact surface between the HTL and the AL, finally resulting in greater performances of the cell. In our case, the roughness shows only limited variation with the stoichiometry, as demonstrated by AFM, thus we infer that the modifications of the interface between the HTL and AL with X are not the main responsible for the variation of the PCE. 

The increase in PCE is mainly linked to an increase in J_sc_ with X, as visible from the J-V characteristic of these devices (Figure 3c). A similar trend was observed by Zhang et al. [32] for OSCs with Mg-doped CuCrO_2_ thin films as HTL, where an increase of J_sc_ was reported for more conductive films, coherently with our findings. In Zhang’s work, the use of 5%-Mg-doped CuCrO_2_ in combination with a non-fullerene AL, PTB7-Th:ITIC, leads to a greater PCE, around 5.2%. The improvement of the device performances can be attributed or to the greater atmospheric stability of this AL when compared to the one used in our study or to the well-controlled atmosphere used for the fabrication of the solar cells. Based on these considerations, we may speculate that with greater control of the atmosphere during the solar cell fabrication and a more stable active layer, we would obtain higher PCEs with the integration of out of stoichiometry CuCrO_2_ thin films. The proposed HTL presents several advantages, for instance, that the modulation of the optoelectronic properties of CuCrO_2_ by a simple tune of the cationic ratio in the initial solution is less sophisticated than extrinsic doping, and that our films are synthesized at atmospheric pressure by a faster method compared to nanoparticles films. Films synthesized by AA-MOCVD are generally characterized by a higher electrical conductivity than nanoparticles ones, in agreement with the values reported for CuCrO_2_ nanoparticles [58]. This may be attributed to a more compacted morphology for thin films deposited by AA-MOCVD. Additionally, the films synthesized by AA-MOCVD are linked to the ITO through covalent bonds, which allows a greater adhesion between the films and the substrate compared to nanoparticle films, synthesized by physical methods.

For X > 65%, films are less transparent and less conductive, thus jeopardizing the OSC performances. Nevertheless, it is important to notice that the PCE measured for CuCrO_2_: 67% OSCs is higher than the CuCrO_2_:60% devices, despite, the former presents a lower FoM than the latter. As reported before, X = 67% is less transparent but its electrical resistivity slightly higher than X = 60%. However, we found an enhancement of the carrier mobility in these Cu_2_O + CuCrO_2_ composite films, which can mitigate the lower optical and electrical properties of the films with X = 67% as HTLs, resulting in similar efficiencies between the CuCrO_2_: 60% and CuCrO_2_: 67% devices. Furthermore, it has to be reminded that the FoM does not take into account the energy band, extremely important in optoelectronic applications. 

The average parameters (J_sc_, V_oc_, R_series_, R_shunt_, Fill factor (FF), and PCE), as well as their standard deviation, were extracted from the J-V curves of all the devices and listed in Table 1. No trend is observed for R_shunt_ and R_series_ of the Fill Factor (FF); these parameters depend on many intrinsic and extrinsic factors, such as the resistances of the used materials, the quality of the interfaces between the layers, the design of the photovoltaic device, and manufacturing defects. The results reported in this work are not sufficient to establish the main responsible for the variation of these parameters, and any speculation would lead to uncertain conclusions.

The best PEDOT:PSS-based and CuCrO_2_:65% based OSC devices were compared and their J-V curves under AM 1.5 illumination are presented in Figure S5 in Supplementary Materials. Despite the good performances achieved by CuCrO_2_:65% (3.1%), the PEDOT:PSS-based solar cell presents a higher PCE value of 5% as reported by the average value in Table 1.

**Figure 4:**
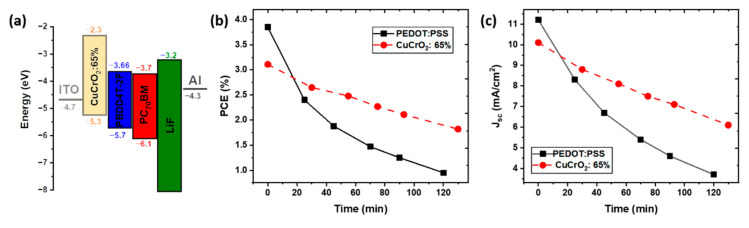
Band diagram of the CuCrO_2_:65% device and stability in atmospheric conditions. (**a**) Schematic energy diagram of the CuCrO_2_: 65% device. (**b**) PCE and (**c**) J_sc_ as a function of the storage time (minutes) in atmospheric conditions at 23 °C, 30% R.H. for OSC based on CuCrO_2_: 65% or PEDOT:PSS.

The schematic energetic diagram of CuCrO_2_:65% is schematized in Figure 4a. The proper energy bands alignment is essential for the conception of OSC in direct configuration. Ideally, the valence band maximum (VBM) must be below the Fermi level of the anode (ITO) and above the HOMO level of the donor material, and the conduction band minimum (CBM) of the HTL is required to be above the LUMO level of the donor material, respectively. This will prevent electron leakage to the anode and ensure the separation of photo-generated holes in the AL.

CuCrO_2_ is generally characterized by a VBM at 5.3 eV [26,59], which guarantees an efficient hole collection at the ITO contact. Its CBM is believed to vary depending on the value of X. Nevertheless, the values obtained by Tauc plot suggest that the CBM is placed above the LUMO of the PBDD4T-2F for all the compositions. Finally, we can assert that the CuCrO_2_ thin films possess well-suited energetic levels to efficiently working as HTL in PBDD4T-2F:PC_70_BM based devices, while preserving ohmic contacts with the ITO electrode, thus representing a valid alternative to PEDOT:PSS [60]. The values of the HOMO and LUMO for ITO, PBDD4T-2F, PC_70_BM, LiF, and Al were obtained from literature [61,62,63].

The stability in atmospheric conditions of CuCrO_2_:65%-based and of PEDOT:PSS-based solar cell was compared. Figure 4b shows the evolution over the time of the PCE of these devices when exposed to open air, with an RH around 30% at room temperature, T = 23 °C. It is noticeable that the PEDOT: PSS-based device has an accelerated degradation of the performances compared to the CuCrO_2_: 65% OSC. Figure S6a shows the normalized values of PCE, easing the quantitative comparison between the two devices. After 120 min in open air, the PCE of the PEDOT: PSS-based OSC is reduced to 25% of the initial value. The integration of CuCrO_2_: 65% leads to a greater lifetime of the device, retaining a PCE around 65% of the original one after more than 2 h under atmospheric conditions. The reduction of efficiency with the time is mainly due to a decrease in J_sc_, Figure 4c, while the V_oc_ is maintained constant over time, reported in Figure S6b in Supplementary Materials. The decrease in current density is more severe in the case of PEDOT: PSS-based devices with a final value around 30% of the original one (Figure S6b in Supplementary Materials). On the other hand, the use of CuCrO_2_ as HTL leads to the conservation of a J_sc_ around 60% of the initial value after 2 h. The loss in short circuit current found for both the devices is attributed to the chemical degradation or morphological evolution of the AL, with many factors contributing to the deterioration of this material [64,65]. These photoactive polymers undergo a phase separation phenomena, leading to a vertical gradient of the acceptor and donor material, significantly impacting the photogeneration process, charge transport, and carrier recombination [66], thus, jeopardizing the short-circuit current. Additionally, it has been reported that photoactive polymers undergo a UV-activated photodegradation process, causing a severe reduction of the photogenerated current [65]. CuCrO_2_ is characterized by a very high absorbance in the UV, as obtained by our optical measurement. This will result in a lower number of photons involved in the photogeneration, limiting the achievable performances. However, we can speculate that the CuCrO_2_ thin films work as a UV-shielding layer, preventing the UV-induced photo-oxidation of the AL, thus resulting in an extended lifetime of the device. 

The faster degradation of the PEDOT:PSS-based device when compared to oxide-based OSCs, is attributed to the deterioration of this polymer through the humidity and oxygen intake; indeed, it has been reported that this material suffers from modification of the morphology when exposed to atmospheric environment, strongly reducing the short-circuit current [17,67]. The faster decay found for PEDOT:PSS-based OSCs can then be explained through the simultaneous degradation of both the HTL and AL. Finally, the integration of out of stoichiometry CuCrO_2_ thin films is highlighted as an auspicious solution to enhance the lifetime of organic photovoltaic devices in atmospheric conditions.

## 4. Conclusions

We reported a chemical vapour deposition technique to synthesize thin films of CuCrO_2_ out of stoichiometry at low temperature and atmospheric pressure. The stoichiometry of the films plays an important role in their structural, morphological, optical, and electrical properties. We have developed deposition conditions in which the synthesis of CuCrO_2_ out of stoichiometry preserves the crystalline delafossite phase, corresponding to a Cu ratio in the starting solution up to X = 65%. These Cu-rich CuCrO_2_ films are characterized by a low electrical resistivity, below 0.1 Ω cm, and a bandgap around 3.0–3.1 eV. It has been found that greater Cu incorporation led to the deposition of nanocomposite films formed of both Cu_2_O and CuCrO_2_. These nanocomposite films have similar electric properties than Cu-rich CuCrO_2_ thin films, but greater hole mobility, close to 1 cm^2^V^−1^s^−1^, and lower energy gap, around 2.9 eV.

We successfully integrated these CuCrO_2_ films with various cationic content as HTL in organic solar cells and the performances of the devices were correlated to the properties of the p-type materials. The highest PCE in this work, 3.1%, was achieved for single-phase CuCrO_2_ synthesized by a Cu precursor ratio of X = 65%, which corresponds to the greatest FoM among the studied candidates. The increase in efficiency was mainly due to an increase of the J_sc_, as result of the trade-off between optical transmittance, electrical properties and band alignment of the HTLs. Furthermore, our design of out of stoichiometry CuCrO_2_ based hybrid solar cells presents a superior lifetime under atmospheric conditions than the organic counterpart. Out of stoichiometry CuCrO_2_ thin films are demonstrated as a promising alternative to unstable PEDOT:PSS, confirming that these p-type TCOs can be successfully integrated into various optoelectronic applications, which require HTL synthesized by a solution-based process at low-temperature.

## Figures and Tables

**Table 1:** Average and standard deviation of the photovoltaic parameters for the different OSCs under 1 sun illumination (AM 1.5G, 100 mW/cm^2^). Reported information are the cationic ratio in the initial solution (X), number of tested devices, short-circuit current density (J_sc_), open-circuit voltage (V_oc_), Fill factor (FF), shunt resistance (R_shunt_), series resistance (R_series_), and power conversion efficiency (PCE) values of the OSCs for each tested HTL.

HTL	Number of Devices	J_sc_ (mA/cm^2^)	V_oc_ (V)	R_shunt_ (Ω cm^2^)	R_series_ (Ω cm^2^)	FF (%)	PCE (%)
PEDOT:PSS	12	10.4 ± 1.0	0.83 ± 0.01	166.9 ± 20.9	6.9 ± 1.6	43.3 ± 3.4	3.8 ± 0.7
X = 40%	6	6.0 ± 0.8	0.74 ± 0.01	202.9 ± 17.0	108 ± 42	39 ± 1.6	1.8 ± 0.3
X = 50%	4	4.16 ± 1.9	0.76 ± 0.01	282.3 ± 91.18	87 ± 67	34 ± 4.7	1.2 ± 0.7
X = 60%	6	8.4 ± 0.6	0.73 ± 0.02	108.6 ± 14.0	46 ± 7	32 ± 2.1	2.0 ± 0.2
X = 65%	12	10.0 ± 0.4	0.75 ± 0.02	113.6 ± 13.3	49 ± 10	37 ± 2.1	2.8 ± 0.2
X = 67%	8	7.6 ± 2.1	0.73 ± 0.02	168.7 ± 80.6	98 ± 55	31 ± 4.4	2.2 ± 0.4
X = 70%	10	6.6 ± 1.3	0.75 ± 0.02	137.1 ± 28.4	83 ± 29	31 ± 8.0	1.9 ± 0.2
X = 80%	4	6.4 ± 0.8	0.77 ± 0.01	166.3 ± 19.5	75 ± 4	34 ± 0.1	1.7 ± 0.2
X = 100%	6	3.7 ± 0.9	0.79 ± 0.01	358.8 ± 76.8	44 ± 17	39 ± 2.7	1.2 ± 0.3

## Data Availability

The data presented in this study are available on request from the corresponding author.

## Supplementary Materials

The following are available online at https://www.mdpi.com/article/10.3390/nano11082109/s1, Figure S1: Structural properties of out of stoichiometry CuCrO_2_ thin films, Figure S2: AFM measurement for out of stoichiometry CuCrO_2_ thin films, Figure S3: Transmittance spectra of the CuCrO_2_ reference for various Cu cationic ratio in the solution, Figure S4: Statistical dispersion of the performances for OSC with out of stoichiometry CuCrO_2_ thin films as HTL, Figure S5: Comparisonof J-V characteristic under A.M. 1.5 illumination of OSC with a HTL of PEDOT:PSS and of CuCrO_2_: 65%, Figure S6: Evolution as function of the time under atmospheric conditions of the performances of the OSC.
