# Supplementary material for: Optimized Stoichiometry for CuCrO2 Thin Films as Hole Transparent Layer in PBDD4T-2F:PC70BM Organic Solar Cells
Source: Nanomaterials (Basel). 2021 Aug 19;11(8):2109. doi: 10.3390/nano11082109 (PMC8398522; doi:10.3390/nano11082109)
Supplement: Supplementary file 1 [file nanomaterials-11-02109-s001.zip › nanomaterials-1301306-supplementary.pdf]

Supplementary Materials

Optimized Stoichiometry for $CuCrO_2$ Thin Films as Hole Transparent Layer in PBDD4T-2F:PC_{70}BM Organic Solar Cells

Lorenzo Bottiglieri [1,*], Ali Nourdine [2], Joao Resende [3], Jean-Luc Deschanvres [1] and Carmen Jiménez [1]

[1] French National Centre for Scientific Research, Laboratoire des Matériaux et du Génie Physique, Institute of Engineering, Université Grenoble Alpes, 38400 Grenoble, France; jean-luc.deschanvres@grenoble-inp.fr (J.-L.D.); carmen.jimenez@grenoble-inp.fr (C.J.)

[2] French National Centre for Scientific Research, The Laboratory of Electrochemistry and Physical-Chemistry of Materials and Interfaces, Institute of Engineering, Université Grenoble Alpes, University of Savoy Mont Blanc-Chambery, 38000 Grenoble, France; ali.nourdine@univ-smb.fr

[3] AlmaScience Colab, Madan Parque, 2829-516 Caparica, Portugal; joao.resende@almascience.pt

* Correspondence: lorenzo.bottiglieri@grenoble-inp.fr

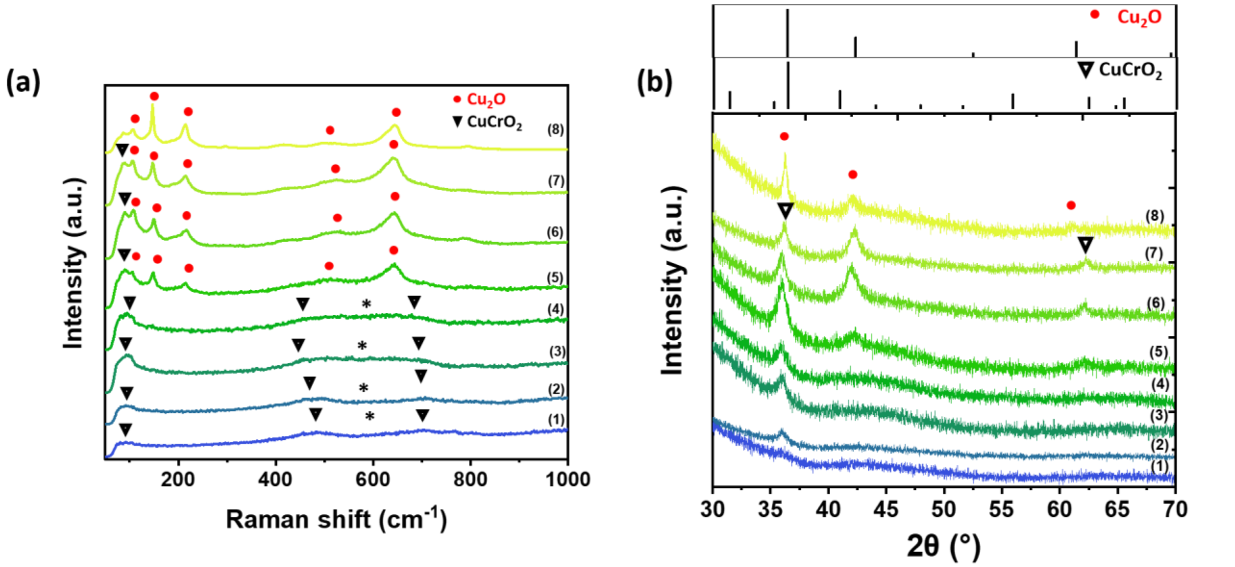

*Figure S1. Structural properties of out of stoichiometry $CuCrO_2$ thin films.(a) Raman spectra of out of stoichiometry $CuCrO_2$ thin films with a Cu cationic ratio in the solution, X, of (1) 40%, (2) 50%, (3) 60%, (4) 65%, (5) 67%, (6) 70%, (7) 80%, (8) 100%. The red dots correspond to Cu_2O Raman modes, black triangles to $CuCrO_2$ ones. Defects related Raman modes are indicated with *. (b) XRD pattern of the same samples. The top windows show the ICDD references of rhombohedral $CuCrO_2$ delafossite structure (space group R-3m, ICDD 04-010-3330), and cubic Cu_2O (space group Pn-3m, ICDD 00-005-0667).*

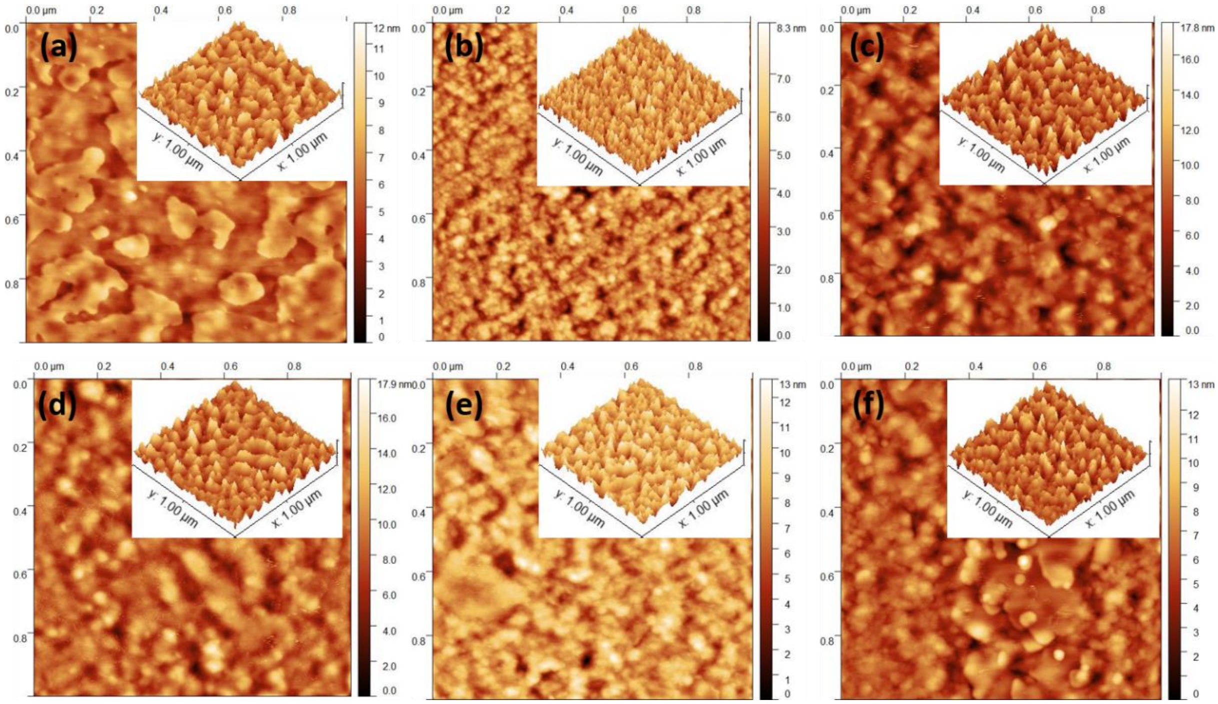

Figure S2. AFM measurement for out of stoichiometry $CuCrO_2$ thin films. Top view of samples synthesized from a solution with a Cu cationic ratio, X, of, (a) 40%, (b) 50%, (c) 60%, (d) 65%, (e) 70%, and (f) 100%. Each inset represent the corresponding 3D mapping of each samples. The measured values of root mean square (RMS) roughness values are 1.3 nm, 1.0 nm, 2.1 nm, 2.0 nm, 1.4 nm, 1.5nm for X of 40%,50% 60%, 65%, 70%, and 100%, respectively.

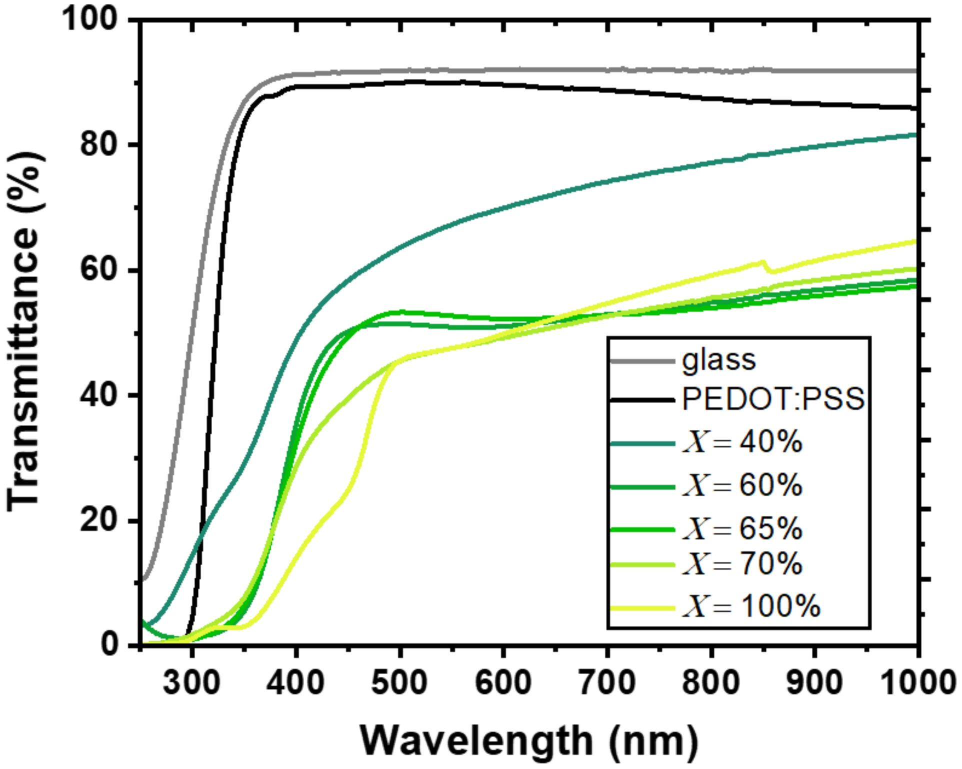

Figure S3. Transmittance spectra of the $CuCrO_2$ reference for various Cu cationic ratio in the solution, X, deposited on corning glass. The substrate, as well as the spin-coated PEDOT:PSS are shown as comparison.

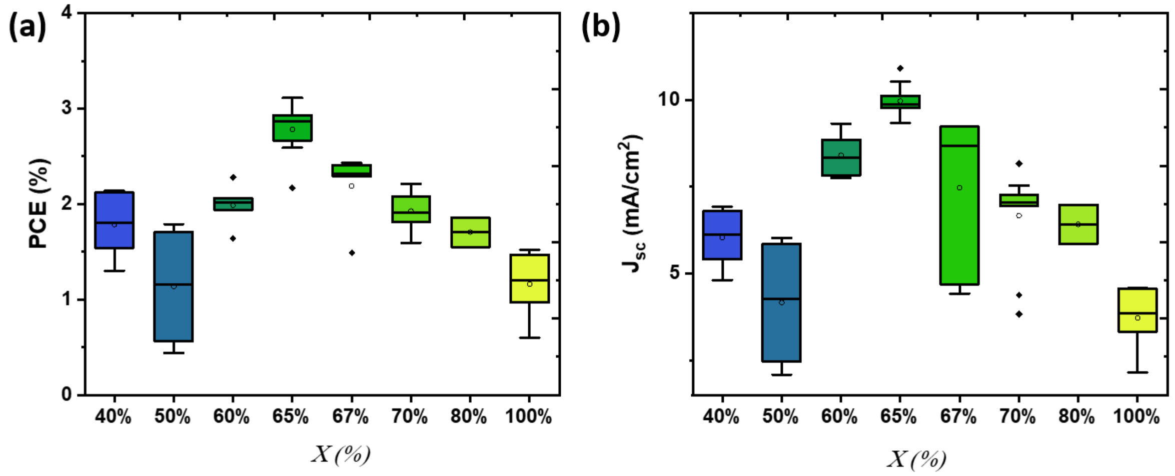

Figure S4. Statistical dispersion of the performances for OSC with out of stoichiometry $CuCrO_2$ thin films as HTL. a) box chart of the PCE calculated from the J-V characteristic under AM 1.5 illumination as a function of the Cu cationic ratio in the solution, X. b) box plot of the variation of the J_{sc} with X. The percentiles are set to 95% whisker top, 75% box top, 25% box bottom, and 5% whisker bottom for each data set.

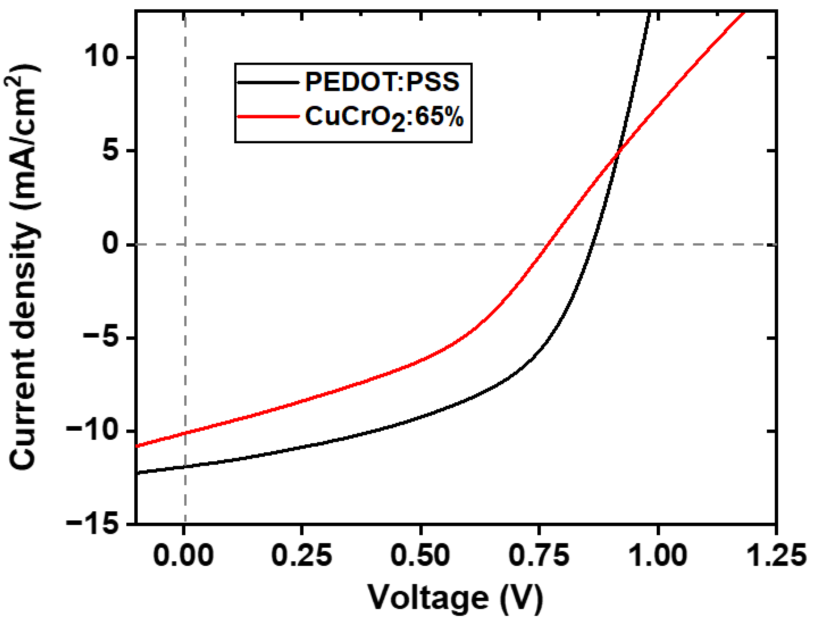

Figure S5. Comparison of J-V characteristic under A.M. 1.5 illumination of OSC with a HTL of PEDOT:PSS and of $CuCrO_2$: 65%.

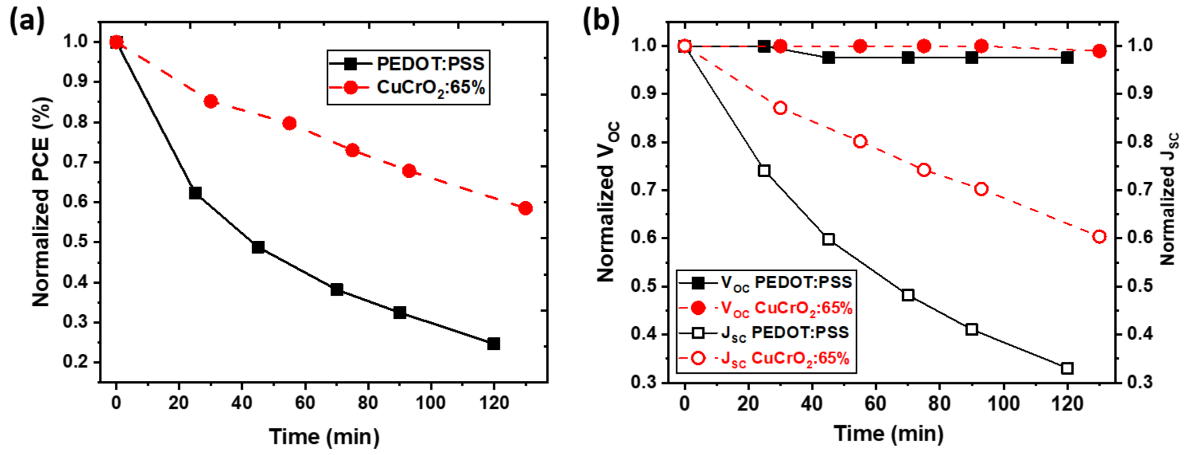

Figure S6. Evolution as function of the time under atmospheric conditions of the performances of the OSC. a) Normalized PCE of PEDOT:PSS-based device (black squares) and $CuCrO_2$: 65% OSC (red circles). b) Normalized open circuit voltage, V_{oc}, (left axis and full symbols) andnormalized short circuit current density, J_{sc}, (right axis and empty symbols) for the same devices. These values are normalized respect the value measured prior exposure to air.
